# A novel adaptive network intrusion detection system for internet of things

**DOI:** 10.1371/journal.pone.0283725

**Published:** 2023-04-21

**Authors:** Parthiban Aravamudhan, Kanimozhi T

**Affiliations:** 1 Electronics and Communication Engineering (ECE), Research Scholar, Vel Tech Rangarajan Dr. Sagunthala R&D Institute of Science and Technology, Chennai, Tamil Nadu, India; 2 Electronics and Communication Engineering (ECE), Associate Professor, Vel Tech Rangarajan Dr. Sagunthala R&D Institute of Science and Technology, Chennai, Tamil Nadu, India; Osun State College of Education, Ila Orangun, NIGERIA

## Abstract

Cyber-attack is one of the most challenging aspects of information technology. After the emergence of the Internet of Things, which is a vast network of sensors, technology started moving towards the Internet of Things (IoT), many IoT based devices interplay in most of the application wings like defence, healthcare, home automation etc., As the technology escalates, it gives an open platform for raiders to hack the network devices. Even though many traditional methods and Machine Learning algorithms are designed hot, still it “Have a Screw Loose” in detecting the cyber-attacks. To “Pull the Plug on” an effective “Intrusion Detection System (IDS)” is designed with “Deep Learning” technique. This research work elucidates the importance in detecting the cyber-attacks as “Anomaly” and “Normal”. Fast Region-Based Convolution Neural Network (Fast R-CNN), a deep convolution network is implemented to develop an efficient and adaptable IDS. After hunting many research papers and articles, “Gradient Boosting” is found to be a powerful optimizer algorithm that gives us a best results when compared to other existing methods. This algorithm uses “Regression” tactics, a statistical technique to predict the continuous target variable that correlates between the variables. To create a structured valid dataset, a stacked model is made by implementing the two most popular dimensionality reduction techniques Principal Component Analysis (PCA) and Singular Value Decomposition (SVD) algorithms. The brainwaves made us to hybridize Fast R-CNN and Gradient Boost Regression (GBR) which reduces the loss function, processing time and boosts the model’s performance. All the above said methods are trained and tested with NIDS dataset V.10 2017. Finally, the “Decision Making” model decides the best result by giving an alert message. Our proposed model attains a high accuracy of 99.5% in detecting the “Cyber Attacks”. The experiment results revealed that the effectiveness of our proposed model surpasses other deep neural network and machine learning techniques which have less accuracy.

## Introduction

In the modern digital world, technology crime becomes “DEVIL” for all people in all domains. Especially, cyber-attacks are increasing drastically due to an open platform of the Internet. Today’s Internet offers ubiquitous connectivity to a variety of devices with various operating systems, which in fact widen the attack surface and introduce a number of new attack vectors. This seems to be crucial issue and hence Network Security is considered as a primary research [1].

Due to the widespread of communication devices and advancement in IoT, the attack surface becomes much easier for attackers to hack the network devices. Network intrusions are unauthorized activities which invade into an organization or a business computer systems which results in data breach and network failure [2]. Passive and closed firewalls find it challenging to provide secure network protection due to the advancement of network technology. Hence IDS is one of the best solutions in detecting the cyber-attacks. An IDS is a device that identifies and collects the information about anonymous activities by monitoring the entire network traffic. It also gives an alert or alarm when any suspicious or any vulnerable activity occurs at the target system [3].

An IDS is primarily classified into two main categories. Host Based Intrusion Detection System (HIDS) pay attention to the running process, monitors the network traffic and audit the system logs. Whereas, Network Based Intrusion Detection System (NIDS) identifies any malicious activity by scanning and tracking down the entire network traffic and triggers an alarm or alert at the time of intrusions [4]. Furthermore, signature-based and anomaly-based are the two types of NIDS that can be identified from the pattern behavior.

Signature-based NIDS detects the known cyber-attacks only if the signature matches that are previously available in the database. Otherwise, it will let on all unknown attacks. Whereas, Anomaly-based NIDS are very efficient in detecting the known and unknown cyber-attacks. But still it needs an efficient optimizer algorithm in-order to analyze and identify the correct cyber-attacks [5].

Many security solutions have been created throughout the years, some of which have proven to be capable of stopping certain attack variations [6]. As the usage of Internet increases very fast, vast volume of data are used as an input for various devices, especially for IoT devices. Most frequent attacks are Denial-of-Service (DoS), Distributed Denial-of-Service (DDoS), Root 2 Local (R2L), User 2 Root (U2R) and Probe etc., All of the aforementioned attacks happen often today. Frequent changes in the behavior of attacks makes tedious in detecting the cyber-attacks [7].

The majority of NIDS research is focused on supervised learning. Authors [8] had done Binary classification as normal and anomaly using Naïve Bayes classifier and achieved an accuracy of 96.5%. Multi-class classification has done by [9] of showing five different types of classes using Artificial Neural Network (ANN) which gives 94.7% accuracy. Using decision tree-based categorization methods, a 97.49% accuracy was attained [10]. Unsupervised learning algorithms are focused very less due to its low performance for NIDS. Expectation–Maximization (EM) clustering, k-medoids, distance-based outlier detection and k-means are some of the unsupervised learning algorithms used for NIDS which shows very less accuracy percentage ranging from 57.81% to 80.15% [11].

Unsupervised clustering methods were used by [11], to create an NIDS that can find abnormalities without labelled data. The author’s classified normal and anomaly instances based on the cluster’s speculation. However the model doesn’t have any threshold value in-order to differentiate between normal and anomaly detection. Either it should be fixed by researcher or it must be fully automated.

Many anomaly detection methods are based on deep learning algorithms to develop an efficient model in-order to detect the cyber-attack effectively. The anomaly detection model is trained from an idealized dataset in which the data’s may be too long and consumes more time. Due to change in the network environment frequently, the model should have the capacity of relearning. If it fails, the performance will decrease [12]. The model is first trained to identify anomalous traffic behavior. Based on the learned behavior the model tries to identify the abnormal traffic in the network [13]. An anomaly detection approach is more effective than a misuse-based detection method since it does not require prior knowledge about an attack to detect it [14].

Although the design of NIDS has not received much attention when using supervised learning algorithm, many researchers deployed this method for various reasons in-order to attain a good result. Principal Component Analysis (PCA), an unsupervised algorithm is deployed by [15], in-order to reduce the dataset dimension. The input variables are preprocessed with increase in time complexity. After preprocessing there are lot of chances for data loss. Singular Value Decomposition (SVD), a type of factorization method retains the original data by occupying very less space [16]. Singular values represent the most energy that can be packed into a data while yet maintaining acceptable consistency even when the data experiences slight distortion. By employing SVD, the suggested method reduces the dimensionality of block vectors and offers the superior attribute of stability [17].

The development in the field of Artificial Intelligence (AI) and Machine Learning (ML) created a major impact in the field of cyber security especially when it comes to research. Specifically the use of Neural Networks for Intrusion Detection System becomes the main focus [18]. Although it has higher accuracy in detecting the cyber-attacks, still it lags in segmenting the packets, eigenvalues calculation and imbalanced dataset [19]. Furthermore, R-CNN, a Deep Convolution Neural Network (DCNN) model identifies the cyber-attacks with high detection accuracy and performance by reducing the False Positives [20].

To identify and detect the cyber-attacks, our proposed method utilizes deep learning algorithm, a subset of machine learning algorithm. Since our dataset has more than 2,00,000 records, deep learning algorithm gives an appropriate solution in analyzing and identifying the pattern behavior during the network traffic. Through this research, we developed an “Efficient Network Intrusion Detection System” which uses a Fast R-CNN model to analyze and learn the behavior of cyber-attacks. The proposed model is hybridized with Gradient Boost Regression which focuses on Denial-of-Service (DoS), Distributed Denial-of-Service (DDoS), Root 2 Local (R2L), User 2 Root (U2R), Probe and Normal. The proposed model is trained and tested on NIDS V.10 2017 dataset to show the performance is more effective when compared to other existing methods.

The rest of the paper is organized as follows: Section 2 explains the related works. Section 3 describes the proposed work in detail. Performance evaluation is briefed in Section 4. Section 5 and 6 explains the experimental results and discussions. Conclusion of the proposed work is briefed in Section 7. Finally Section 8 ends up with Challenges, limitations and future scope of the research work.

## Related works

Nguyen, M. T and Kim, K [21] designed a Genetic Convolutional Neural Network for Intrusion Detection System. An Improved Feature Subset (IFS) is utilized, a combination of CNN extractor and BG classifier to build an efficient system using Genetic Algorithm. With the help of multiple layers, the extractor produces an excellent with standable Deep Feature Subset (DFS) which are learned by the peculiar pattern. The 5-fold CV process utilizing the DFS as the input was then used to verify the performance of the BG classifier. The model’s detection performance is improved by constructing a 3 layer features, a combination of Genetic Algorithm (GA), Fuzzy C-Means clustering (FCM) and CNN by identifying the peculiar features. Moreover the proposed model’s performance is increased when applying 5-fold CV in all phases which also reduces the overfitting problem. NSL-KDD dataset is deployed to test the performance of the proposed model. The model achieves only 97% Accuracy in binary classification and 98% in multiclass classification which should be considered as one important factor.

Shisrut Rawat et al. [22] implemented and evaluated Intrusion Detection System using Deep Neural Network. They created 5-layers Deep Neural Network (DNN), an iconic Feed-Forward Network (FFN) which contains an input, output and many hidden layers. The authors implemented PCA, a dimensionality reduction technique to create a standardized dataset. In-order to avoid vanishing gradient problems they introduced “Relu” an activation function which removes the gradient error. Simultaneously they used “Sigmoid” activation function at the output layer. This method works on binary cross-entropy loss function which throws the output as either “0” or “1”. The model is trained and tested using NSL-KDD dataset on GPU enabled Tensor flow and compared the performance with all features and 6 SDN features. The proposed model shows 96% as training accuracy and only 79% as testing accuracy with PCA+DNN. This model has a huge drawback during testing. Due to less accuracy the model’s performance is also very weak. Performance is also an another important factor that should be considered.

Yin et al. [23] introduced the Recurrent Neural Network-Intrusion Detection System (RNN-IDS) model as a robust and reliable framework for assessing network security. It had strong modeling ability for intrusion detection. The RNN-IDS model performed better than other methods when it comes to accuracy, detection rates and false positive rates. This methodology functions better for multi-class classification for NSL-KDD datasets. The RNN-IDS model was able to detect different types of intrusions. This method has a huge drawback that the model has not trained and tested in GPU. It consumes more time during training session. Due to this, the model faces problems like acceleration, exploding and vanishing gradient problems.

Jaswinder Singh and Dr. B.K.Sharma [24] designed R-CNN Based Object Detection and Classification Methods for Complex Sceneries. In-order to achieve high detection and classification accuracy, authors have merged the existing feature extraction with shape feature extraction technique. They tested the model on VOC 2007 dataset which is publically available. The authors utilized “Relu” and dropout layers in-order to overcome the overfitting problem. The proposed model uses “CovNet” for selective search. The proposed model shows very less mean, Average and Precision (mAP) in detecting complex objects, which ranges from 50% to 80% only. The model shows that detecting accuracy is very less and the efficiency is also drastically reduced. From the results we clearly understand that an efficient optimizer algorithm is essential to increase the detection rate, accuracy and efficiency.

Sydney M.Kasongo and Yanxia Sun [25] analyzed and tested the performance of intrusion detection systems using a feature selection method. The authors built their model using machine learning techniques. Data preprocessing is done with cleaning, normalization and feature selection. XG Boost algorithm, a filter-based method is implemented to reduce the loss function and also extracts the prominent features required for training and testing. To overcome the computational issue, Z-score standardization is done. The model is trained and tested on UNSWNB-15 dataset to measure the performance of the model. The results show that this model is very weak in all the four metrics (Accuracy, Precision, F-Measure and Recall). From the results we identified that deep learning gives us best results when compared to other machine learning models.

Martin Sarnovsky and Jan Paralic [26] designed a hybrid knowledge based and machine learning based approach. They proposed this method to identify the severity of new attack types and also the existing network attacks. They introduced hierarchical classification which are divided into two phases. In the first phase, the attack detection model predicts the attack on the high-level of pecking order which classifies as “Attack” and “Normal”. In the second phase, the attack is identified based on the label and categorized according to classes of attacks. Weighted voting scheme is implemented to identify the final prediction on the basis of performance classification. The performance of the model is trained and tested on KDD 99 dataset. This model fails to identify and detect new attack types at the right time. So this model has to be retrained again to detect the future attack, which is a huge drawback.

Muhammad Almas Khan et al. [27] designed a deep learning based intrusion detection system for MQTT enabled IoT. The performance of the model is trained and tested on MQTT-IoT-IDS2020 dataset. The proposed model follows forward and back propagation for preprocessing method. Sigmoid activation function is implemented to avoid biasing and softmax classifier is implemented to reduce the loss function of the model. Cross entropy method is applied to classify the output as attack and normal. ADAptive Moment estimation (ADAM) optimizer algorithm is the key factor to reduce the cost function. The proposed model is compared with various machine learning algorithms which are Naive Bayes (NB), Random Forest (RF), K-Nearest Neighbor (KNN), Decision Tree (DT), Long Short-Term Memory (LSTM), and Gated Recurrent Units (GRUs). For all network features (packet-flow, bi-flow and uni-flow) the accuracy seems to be less (90.7%, 98.12% and 97.08%) respectively in-terms of multi-class classification. The other performance metrics are also seem to be less in this model. The proposed model fails to identify the known attacks, new attacks and vulnerabilities present in different protocols of IoT.

Omar A. Alzubi [28] introduced A deep learning- based frechet and dirichlet model for intrusion detection in Industrial Wireless Sensor Network (IWSN), to increase the detection rate and to reduce the detection time. The Frechet Hyperbolic Deep and Dirichlet Secured (FHD-DS) model identifies the anomaly pattern that deviates from the actual detection. The traffic and relevant data’s are obtained from each input layer with added weights. The error rate is calculated and reduced using derivative gradient vector. The Frechet Hyperbolic Deep Traffic (FHDT) algorithm utilizes reverse error assessment process and exponential hyperbolic non-linear function to extract the inherent traffic features and the network related activities. Statistical dirichlet Anomaly-based Intrusion Detection System (SD-AIDS) is implemented to learn the traffic features. The learned activity increases the performance of the model, by calculating Processing Time (PT), Response Time (RT) and Accelerating Rate (AR). The number of successful and failed transmissions are calculated by Sensor Transmission Caliber (STC). The proposed model is trained and tested on KDD Cup’ 99 dataset to measure the performance. The proposed model attains 90% Intrusion Detection Rate (IDR) and 92% in data delivery rate. The proposed model shows that it is weak in identifying and detecting the cyber-attacks correctly and efficiently and also loss of packets are high.

Sugandh Seth et al. [29] designed a novel time efficient learning‑based approach for smart intrusion detection system. A valid dataset is formed using data wrangling technique. The filter and wrapper method is embedded to select the prominent features and to calculate the performance of the model. PCA and RF is used to reduce the dataset dimension. After removing the redundancy and irrelevant features, PCA reduces the prediction latency which results in high accuracy. The Hybrid feature selection, which reduces the number of input features while maintaining the crucial data, is implemented to reduce the prediction lag. The power, speed and performance of the model is increased by implementing Light Gradient Boost Machine (GBM). To identify the predicted class based on the distance between original data and the predicted data, K-Nearest Neighbor (KNN) classifier is used. The performance of the proposed model is calculated on CIC-IDS 2018 dataset. Even though the precision and specificity seems to be 99.3% and 99.4% in this model, the accuracy, F-measure and recall seems to be lagging when compared to other existing methods.

From literature study, major research gaps are identified to design an efficient IDS model. Selecting the hyper-parameters carefully makes the model perfect. Accuracy, Time Consumption, Detection Rate, Performance Metrics and Efficiency are still to be improved. Simultaneously cost and time for training and testing are still to be reduced. In addition to that, a network environment must be considered as a main criteria to design an efficient IDS. In-order to quickly identify and detect the cyber-attack the proposed system should have the ability to send the logs and reports to the monitoring server.

By quickly considering the above all aspects, an Efficient IDS is designed in detecting the cyber-attacks. Our proposed work outperforms other existing methods.

## Proposed methodology

### Input dataset description

The NIDS dataset was created which are simulated in a typical US Air Force LAN to establish a setting for acquiring raw TCP/IP dump data for a network. The network environment was designed and named as “Label” to identify the features as “Anomaly” (or) “Normal”. The labelled features are then classified as “DDoS”, DoS”, U2R, R2L, Probe and Normal. The Train Data set contains 25192 rows × 42 columns and the Test Data set contains 22544 rows × 41 columns. The dataset contains totally of 41 features of which 38 are quantitative features and 3 are qualitative features. The data’s are collected through Transmission Control Protocol/Internet Protocol (TCP/IP) protocol from source IP address to destination IP address. “Basic”, “Content” “Time” and “Host” are the 4 primary features that are divided into groups, based on the characteristics. Table 1 shows the dataset characteristics and records of NIDS dataset V.10 2017. The dataset “Network Intrusion Detectiom” ia available online at: https://www.kaggle.com/datasets/sampadab17/network-intrusion-detection which can be accessed directly [30].

**Table 1:** Data repository.

**Category**			**Category**		
**Basic Features**	No.	Attribute Name	**Content Related Features**	No.	hot
	1	duration		10	num_failed_logins
	2	protocol		11	logged_in
	3	service		12	num_compromised
	4	flag		13	root_shell
	5	src_byte		14	su_attempted
	6	dst_byte		15	num_root
	7	land		16	num_file_creations
	8	wrong_fragment		17	num_shells
	9	urgent		18	hot
**Category**			**Category**		
**Content Related Features**	No.	Attribute Name	**Time Related Features**	No.	Attribute Name
	19	num_access_files		23	count
	20	num_outbound_commands		24	srv_count
	21	is_host_login		25	serror_rate
	22	is_guest_login		26	srv_error_rate
				27	rerror_rate
				28	srv_rerror_rate
				29	same_srv_rate
				30	diff_srv_rate
				31	srv_diff_host_rate
**Category**					
**Host Based Traffic Features**	No.	Attribute Name			
	32	dst_host_count			
	33	dst_host_ srv_count			
	34	dst_host_same_srv_rate			
	35	dst_host_diff_srv_rate			
	36	dst_host_same_src_port_rate			
	37	dst_host_srv_diff_host_rate			
	38	dst_host_serror_rate			
	39	dst_host_srv_serror_rate			
	40	dst_host_rerror_rate			
	41	dst_host_srv_error_rate			

#### Complete network configuration

Firewalls, Routers, Switches, Modems and the existence of Windows operating systems make up a full network topology.

#### Packet protocols

Internet Control Message Protocol (ICMP), TCP/IP and User Datagram Protocol/Internet Protocol (UDP/IP).

#### Anomaly-based protocols

Here, an IDS has been configured to identify irregularities in protocols that are generally said to be an attack. If a particular protocol has more than 50% “Packet Loss” during normal operation, the proposed model gives an alert. This happens only when the detected norms differ from the packet loss percentage.

#### Data flow analysis

When there is a sudden change in transmitting a large amount of data, the proposed model examines the data flow across the network and pinpoints the location of any anonymous issue. The raw traffic were captured using the TCP/IP dump tool.

#### Preprocessing

The preprocessing of the input dataset consists of reading the data, resizing the dataset and removing the junk values, unknown characters etc., from the raw dataset. Our proposed model uses NIDS dataset V.10 2017 which has more than 2,00,000 data’s available as.CSV file.

Initially we are generating a feature matrix “X” and an observation vector “Y” with respect to “X” to find out any missing values. These missing values are estimated by calculating “Median” “Mean” and “Variance”. Then our learned class “Label.Encoder()” will convert the “categorical values” into “numerical values”.

To avoid “biasing” problem and to reduce the training cost, “feature scaling” should be done. To narrow down the data values “Scale to Range” method is proposed in our work. To regularize and to set the data in a similar scale, “Normalization” is done to rescale our data between of “0 to 1”. This method helps in finding the data losses and outliers in the proposed model.

To reduce the size of the dataset “PCA” algorithm, a dimensionality reduction technique is proposed to minimize the residuals, information loss and overfitting. The “variance” and “performance” of the proposed model is maximized after principal values are achieved.

To obtain singular values in the dataset “SVD” algorithm, a matrix decomposition technique is implemented. Eigen vectors are calculated to find the Eigen values of the matrices. The square roots of each Eigen values which is greater than zero are considered to be “Singular values”.

As a result of doing the above methods, a new structured dataset is created. Finally, our dataset is divided into 70% → “Training set” + 30% → “Testing set”, an ideal proportion to train and test our proposed model.

**Fig 1:**
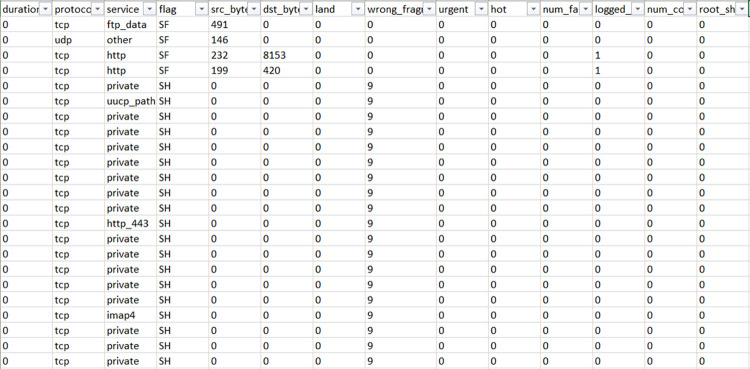
Trained dataset after preprocessing.

Fig 1 shows the screenshot of the NIDS Dataset under training. The dataset is preprocessed for the further analysis during the training session. The type of protocol utilized, type of service, flag status, byte sent from source and received by the destination are considered important for the analysis.

**Fig 2:**
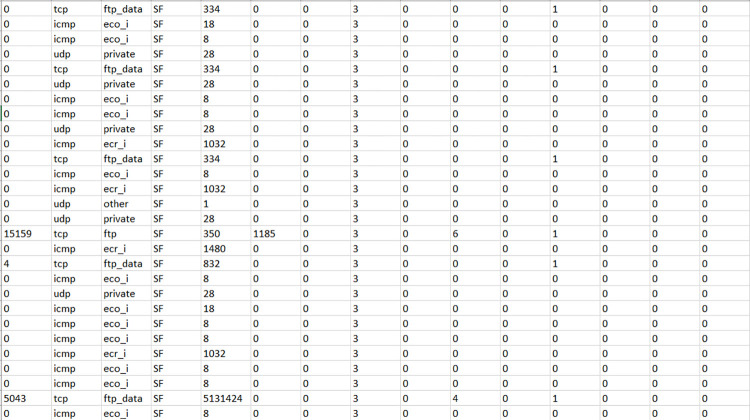
Test dataset after preprocessing.

Fig 2 shows the screenshot of the NIDS Dataset under test. The dataset is preprocessed for the further analysis during the testing session. The type of protocol utilized, type of service, flag status, byte sent from source and received by the destination are considered merely important for the analysis.

#### Feature extraction of NIDS

**Fig 3:**
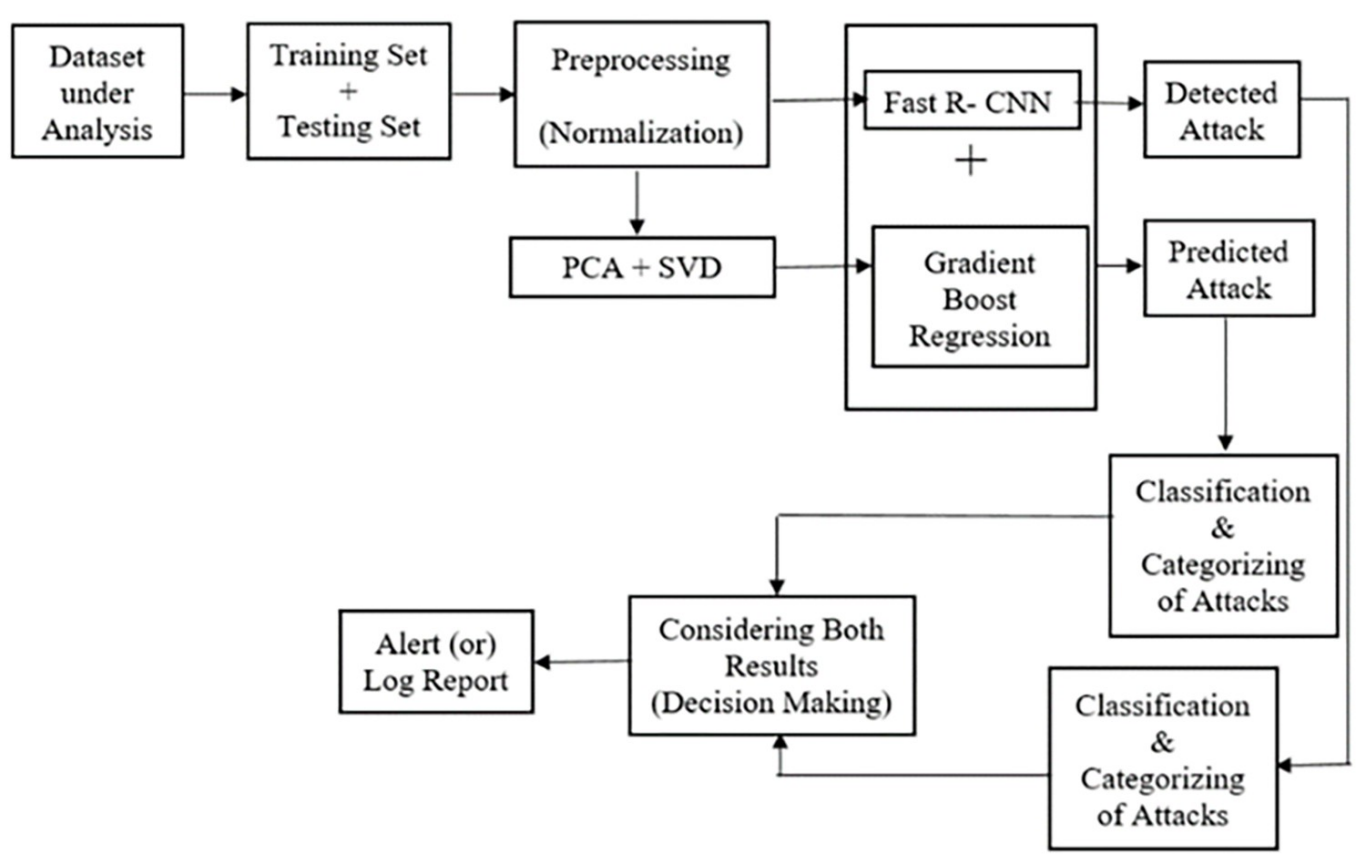
Feature extraction of proposed NIDS.

**Fig 4:**
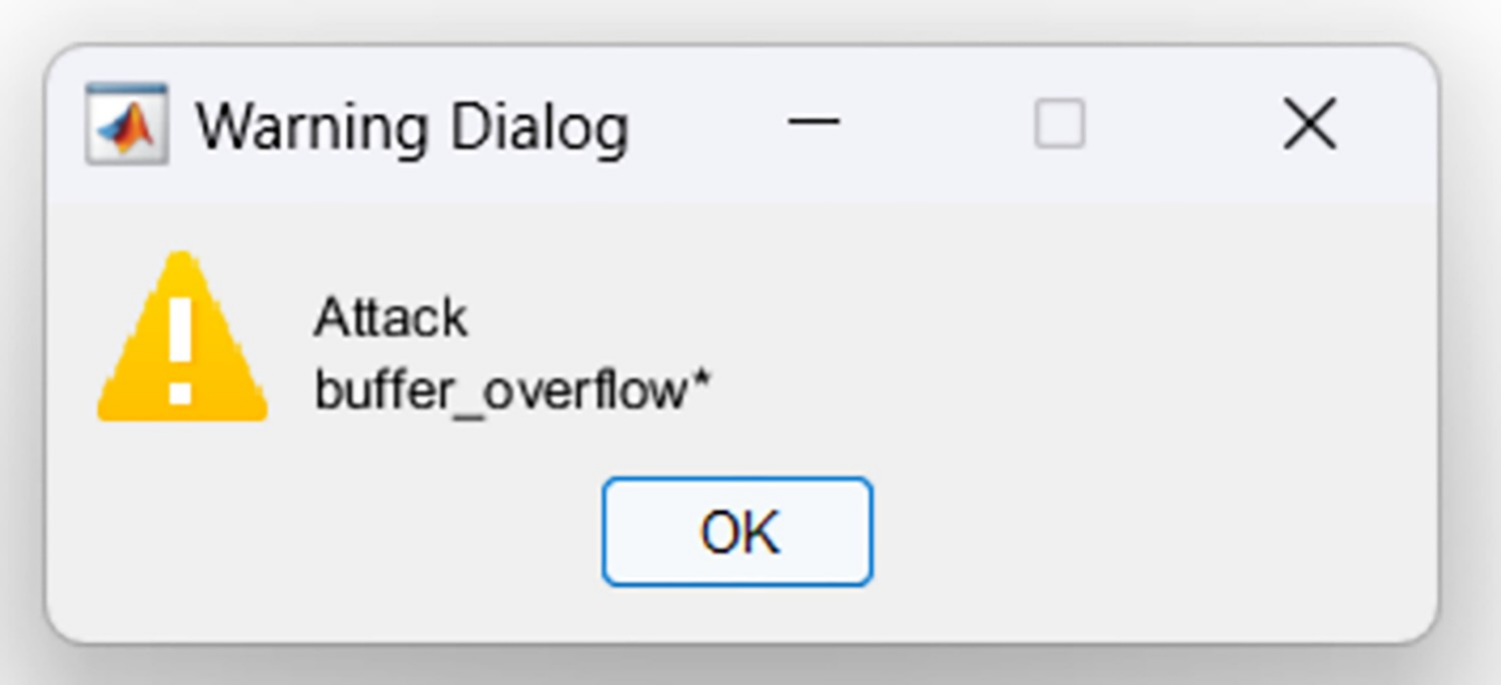
Warning dialog box of proposed NIDS.

Our proposed method uses NIDS dataset V.10 2017 to train and test the model. Our dataset is preprocessed by applying Normalization technique which converts the set of data into a range of scale. After the features are extracted, it is fed into Fast R-CNN, which is a pre-trained model. This model identifies and detects the cyber-attack based on the anomaly pattern generated. At the same time, simultaneously, our proposed method uses PCA to reduce the dimensionality of data. Another method SVD, a matrix factorization technique decomposes several matrices into original matrix. After the features are extracted from the dataset, it is given as an input to gradient boost regression combined with Fast R-CNN, a hybrid model to learn the features very fast and also to boost the performance of the proposed model. Based on the pattern generated, our proposed hybrid model detects the cyber-attack from Fast R-CNN architecture. The output are then classified as “Normal” or “Anomaly” as binary-class classification and further categorized as “R2L, U2R, DoS, DDoS and Probe as multi-class classification. Simultaneously, our proposed stacked model predicts the cyber-attack from gradient boost regression hybridized with Fast R-CNN. The output are then classified as “Normal” or “Anomaly” as binary-class classification and further categorized as “R2L, U2R, DoS, DDoS and Probe as multi-class classification. Our proposed model now compare both the results and decides the best detected cyber-attack through a “Decision Making” block which is shown in Fig 3. Finally our proposed NIDS model gives a warning dialog box as “Buffer Overflow” as attack detected which is shown in Fig 4.

#### Principal Component Analysis (PCA) for network intrusion detection system

To identify the principal components present in the dataset, a dimensionality reduction technique called PCA is utilized in our proposed work. This method not only reduces the dimension of the dataset, but also retains the maximum number of information. At first the standard regression co-efficients are calculated. Once the matrix is reduced, the “Regression co-efficient” which has only particular features exceeds a threshold “T_h_” value, then it is detected as “ATTACK”. This Threshold value is calculated using Cross-validation. If the particular features do not exceeds the “T_h_” value then the value is detected as “NORMAL”. Eq 1 represents the condition for the “Cyber-Attack”.

$$\left\{\begin{array}{l} d(x)>T_{h}:\;attack\\ d(x)\leq T_{h}:\;normal \end{array}\right\}$$ (1)

Where “T_h_” denotes “Threshold Value”.

Before performing PCA, normalize the data into standard dataset. We should also ensure that the Mean should be “0” and Variance should be “1”. This can be achieved by “Min-Max” scaling technique, by doing shifting or rescaling.

$$X’=\frac{X-Xmin}{Xmax-Xmin}$$ (2)

Where, Xmax and Xmin are Feature values.

After rescaling is done, we have to compute “Covariance Matrix (p × p)”. Where “p” is the number of dimensions. This matrix understands the correlation between two or more features. That is, how the variables of the input dataset are varying from “Mean” with respect to each other.

$$Covariance\;Matrix=\left[\begin{array}{cc} Cov(X,X) & Cov(Y,X)\\ Cov(Y,X) & Cov(Y,Y) \end{array}\right]$$ (3)

$$Covariance=\frac{Sum(X-(Mean\;of\;X))(Y-(Mean\;of\;Y))}{Number\;of\;Data\;points}$$ (4)

Where “X” and “Y” are 2 Dimensional dataset with 2 variables

In order to compute the covariance matrix, we need to define the various elements of it, such as the "Eigen Vectors" and "Eigen Values”. These two components are then computed by the special variable for analysis SVD. The order of the "Eigen Values" and "Eigen Vectors" is taken into account to identify the principal components of the matrix.

After identifying the principal components, the feature vectors are calculated to form a Matrix that contain “1s” only. These feature vectors are isolated by identifying the lesser significance (of low “Eigen Values”).

The normal Scrutiny is defined as

$$\mu =\frac{1}{n}\sum_{i=1}^{n}x_{i}$$ (5)

The deviation from the average is defined as

$$\phi_{i}=x_{i}-\mu$$ (6)

Once the “Feature Vectors” are calculated, the featured data are reshaped along the “Principal Component Axes”, using the “Eigen Vectors” of the covariance matrix. The final dataset is calculated by applying the following formula,

$$Final\;Dataset=Feature\;Vector^{T}\times Normalized\;Original\;DataSet^{T}$$ (7)

By performing all the above steps, our final NIDS dataset is created for further process. It maximizes the “Variance” of the data and minimizes the “Residuals” (Squared distance). It increases the interpretability of the model. (Understanding in-terms of humans). It also minimizes the “Information Loss” and mitigates the “Overfitting” problem. Finally our proposed model’s performance is increased at a very low cost [31].

#### Singular Value Decomposition (SVD) for PCA

This method can transform correlated variables into uncorrelated ones by exposing the relationships among the data elements. The segment of grid **“A”** that’s at line i and line j is referred to as A[ij]. If **"B"** is a "**p×n**" network and "**A**" is a "**m×p**" matrix, then the product of two matrices is "**C = AB**" must contain “**m×n**” shape. The final matrix “C” can be calculated using the formula,

$$[C_{ij}]=c_{ij}=\sum_{k=1}^{p}a_{ik}b_{kj}$$ (8)

The matching row and column vectors in matrices A and B are dot products of product matrix “C”. The square roots of the values of "C" which are greater than zero, represent the singular ones [32].

### Gradient boosting algorithm for network intrusion detection system

The most authoritative optimizer algorithms in Machine Learning techniques is “Gradient Boosting” algorithm. Whenever a model tries to learn the data, probability of occurring errors are more. As we know that “Bias Error” and “Variance Error” are the two errors occurring in the Deep Learning method. So “Gradient Boosting” is one of the best solutions to minimize the “Bias Error” of the proposed model. To minimize the “Variance Error” or “Residuals” new model is built on the errors of the previous model.

The proposed optimizer algorithm utilizes “Regression” method as our target column in the NIDS dataset which has continuous values. That is either “Anomaly” or “Normal”. Then based on the pattern generated it classifies the cyber-attack. “Loss Function” is a major difference between them. To mitigate the “Loss Function”, weak learners are added to make the model strong using “Gradient Descent” method. Our proposed model utilizes “Gradient Boost Regression” which is based on the loss functions. It is calculated by “Mean Square Error (MSE)”. By reducing the MSE further, our proposed model’s efficiency is increased.

The initial step in gradient boosting is building a base model to predict the observations in the training dataset. To make things simple, we average the target column and take it as the forecast number. This can be calculated by using the formula,

$$F_{o}(x)=\arg_{\gamma }\;\min\sum_{i=1}^{n}L(Y_{i},\gamma )$$ (9)

Here, “F_0_” is the constant value prediction, “L” is the loss function, “Y” is the target value and “γ” is our predicted value. argmin helps in calculating the minimum loss function by calculating the predicted value / gamma. The loss function can be calculated by using the formula,

$$L=\frac{1}{n}\sum_{i=0}^{n}(Y_{i}-\gamma_{i}{)}^{2}$$ (10)

Here, “Y_i_” is the observed value and “γ” is our predicted value. To make this loss function minimal, we have to find the minimum value of gamma. This can be calculated by using the

formula,

$$\frac{dL}{d\gamma }=\frac{2}{2}(\sum_{i=0}^{n}\left(y_{i}-\gamma_{i}\right))=-\sum_{i=0}^{n}\left(y_{i}-\gamma_{i}\right)$$ (11)

Once the minimum value of gamma is obtained, we have to calculate the “Pseudo Residuals”. That is (observed value–predicted value). The predicted value is calculated from the previous model based on the prediction obtained.

On the basis of these “Pseudo Residuals”, we will create a model and make predictions in-order to increase the prediction power and also to improve our proposed model’s accuracy. Now assuming this “Residuals” as a target our model generates new predictions which are nothing but “Error Values”.

By tuning the “Residuals” further, the output values of each leaf are determined by “Decision Tree (DT)” method. This method reveals the exact output by taking average of all numbers in the leaf. The average can be calculated by using the formula,

$$\gamma_{m}=\arg_{\gamma }\;\min\sum_{i=1}^{n}L(Y_{i},F_{m-1}(x_{i})+\gamma h_{m}(xi))$$ (12)

Here h_m_(x_i_) is “DT” on residuals and “m” is the number of “DT”.

The output value of a leaf is known as the gamma value, which helps minimize the loss function. On the left, the "Gamma" represents the output of a specific leaf. The predictions made in the previous model are then updated and the final result is presented. This can be calculated using the formula,

$$F_{m}(x)=F_{m-1}(x)+v_{m}h_{m}(x)$$ (13)

Where “m” represents the number of DT’s created.

Now, to build a new DT we have to calculate new predictions. This can be calculated using the formula,

NewPrediction=Previousprediction+learningrate×thetreemadeonresiduals (14)

Here F_m-1_(x) represents the previous prediction and the learning rate is taken as 0.0001 in our proposed work. Long-term accuracy is increased because it lessens the influence that each tree has on the outcome of the prediction.

Now the new prediction F_1_(x) is calculated from the previous predictions of F_m-1_(x). This process is iterated repeatedly till our proposed model reaches the negligible loss.

Reducing the False Positives is one of the important tasks in our proposed method. Here we calculated “False Positive Error Probability” and “False Negative Error Probability”. After the probabilities are calculated, a new weight is built to calculate every regression’s suggestion by analyzing the probabilities and errors. The new weight can be calculated as

$$\beta =\log(\frac{1-\omega_{t}}{\omega_{t}})$$ (15)

ω_t_ represents the “Total Error”. The previously calculated probabilities are taken into account when computing the new beta, known as "probabilistic beta (β’)”. This can be calculated using the formula,

$$\beta '=[\log(\frac{1-\omega_{t}}{\omega_{t}})]X(\frac{1-P_{FN}}{P_{FP}})$$ (16)

Where “P_FN_” and “P_FP_” are Error Probabilities of False Negatives and False Positives. The False positive error probability is inversely proportional to proposed beta. When there is increase in high-positive error rate in regression, simultaneously the weights are lowered. If it is not, then it is true. Once the weak regression produced high “P_FP_”, the best weak classifier is given a little weight in order to create a strong classifier that decreases the amount of false positives. In contrast to the original approach, the probabilistic beta on updating weight allows for a higher update from incorrectly categorized data. This is due to the fact that the suggested beta will always be higher than the initial beta. Thus our proposed method reaches high accuracy for weak classifier, by applying higher weight to the misclassified data [33].

### Proposed architecture for network intrusion detection system

Ross B. Girshick’s Region-Based Convolution Neural Network (R-CNN) uses the selective search approach to scale the ROI (Region of Interest) and extract features from the target pictures. R-CNN involves forward calculation for numerous region candidates, some of which may overlap [34]. Instead of extracting each region’s image multiple times, Fast R-CNN uses a feature extraction tool to extract the entire image. This process significantly cuts down on the processing time. [35].

The text inputs are identified by the conventional method at character level. Now, the text characters which are cropped from the region are preprocessed and recognized. Text recognition techniques that do not require character segmentation are known as Deep Convolution Neural Network (D-CNN). This technique is based on multi-digit number classification [36].

We choose the text-type bounding boxes depending on the text class after the text positions have been identified. As a text recognizer we implemented Fast R-CNN model in our proposed work. First, from the preprocessed text region, the convolutional layers extract the feature maps. The feature maps are used to extract a series of feature vectors from left to right [37].

Instead of using the other more prevalent models implemented in security-critical systems, the majority of cyber-attack approaches concentrate on image classifiers. This research focuses on detecting the cyber-attack on Fast R-CNN, a model for object detection.

**Fig 5:**
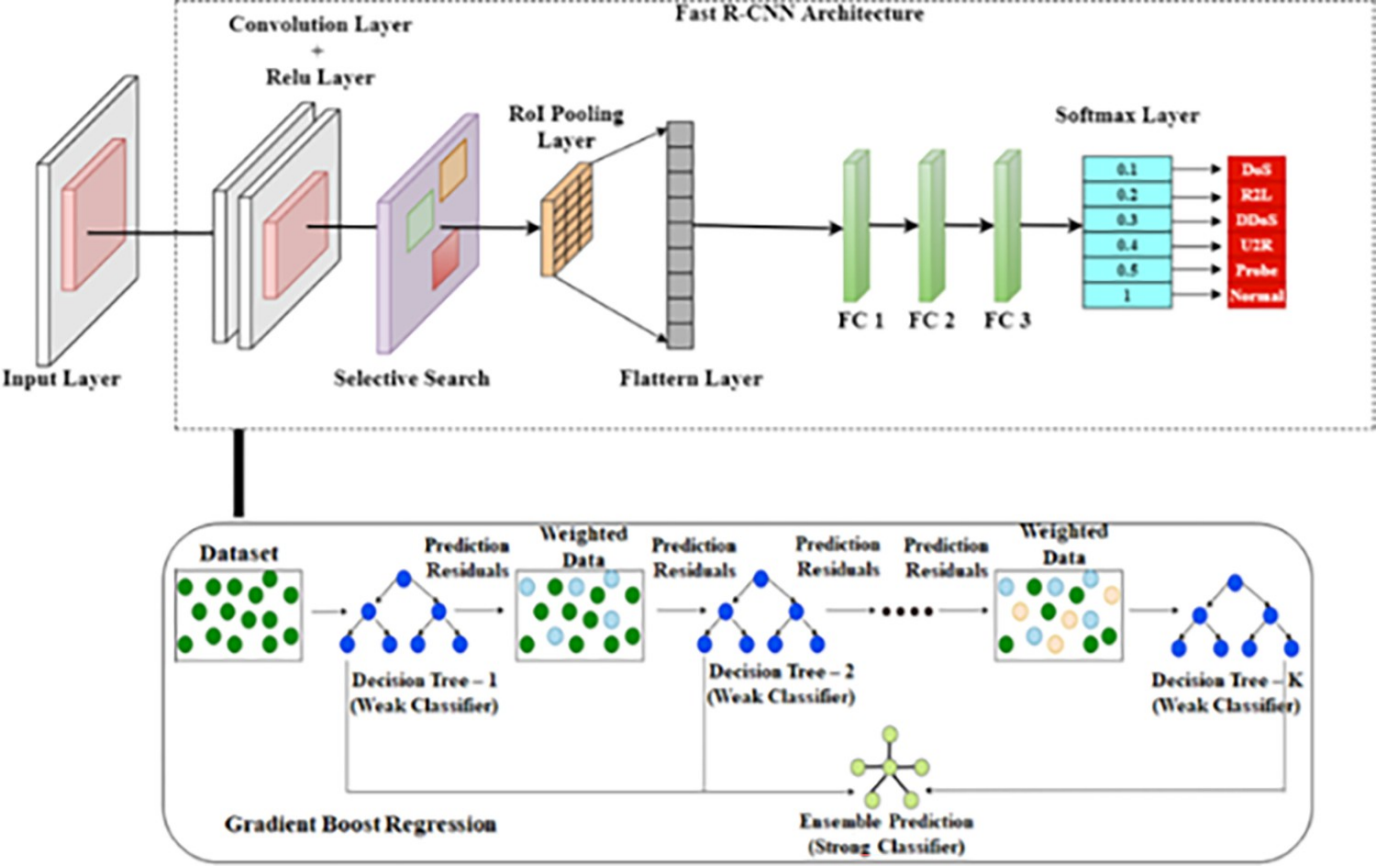
Proposed architecture of fast R–CNN.

To produce the system model as a novel architecture shown in Fig 5, the proposed system is composed of two robust function cascaded for decision making. The DLN model consists of [1000x1x1] input layer, followed by [1x10] of two dimensional convolution layer. Two levels of stacked fully connected layers of size [384] features finally connected with fully connected layer of size [6] with the soft-max layer and classification layer of final stage. The secondary model uses GBR that analyze the input feature vectors and form the relativity graph as scatter plot. The final decision model is created with the help of these two results.

The optimal parameters for training a model are represented in Table 2. When choosing a learning algorithm, it is important to find a set of hyper-parameter values that are ideal for the program. This tuning algorithm can improve the model’s performance and reduce its loss function.

**Table 2:** Optimal hyper–parameters to train the proposed model.

Optimal Hyper-parameters for our Proposed Model	
**Train–Test split ratio**	70%—Training Set 30%—Testing Set
**Learning Rate**	0.0001
**Momentum**	0.9000
**Optimization Algorithm**	Gradient Boost Regression
**Algorithm Method**	Stochastic Gradient Descent (SGD)
**Activation Function**	ReLU
**Modeling the Dataset**	Loss Function
**No. of Activation Units used**	2
**No. of Iterations (Epochs) in Training**	50 to 1000
**Batch Size**	128
**Verbose Frequency**	50
**Drop Out Layer**	2
**Classifier**	SoftMax

Fast R-CNN will use a multi-task loss function to incorporate object recognition and position correction into a single network. To store the data for the features used in the network’s training process, step-by-step training does not need a lot of memory. The Fast R-CNN detector may fail to detect some features and also there is a chance of detecting false features due to some similarity pattern of cyber-attack. So detected features which are obtained by Gradient boost Regression training, will be a good residual detectors. The training speed is increased by Normalization function. Weights, offsets and loss functions are corrected and calculated concurrently when the operation of the network is speeded up by multitask loss function.

The distributed K+1 sample weights of each RoI pooling layer and the discrete probability values output are calculated from the “Softmax” classification layer. The bounding box predict can output the offset value of the regression in the box and is used to modify the coordinates of the candidate text location. The precise loss value is calculated with the help of loss bounding box and the multitask loss function is the loss function of “Softmax” layer.

CNN came into the attention of many academics after the AlexNet model emerged in 2012, and CNN also had a significant impact on the text recognition industry. The existing sliding window operation is utilized for target detection. Feature values are manually annotated after the mesh text has been captured. Then the whole text is passed through to locate the target object by iterating continuously. The feature extraction accuracy is important for Gradient Boost Regression to identify the target detection accuracy. Utilizing ROI in the search area improves the detector’s accuracy.

#### Method for residual detection

The extracted features may contain some incorrect values, missing values or outliers during the time of experimental analysis. Due to these reasons, the accuracy level of the target object detection is reduced and it is not convinced. In order to increase the target object’s detection accuracy, the residual detection approach is applied in this research. Gradient Boost Regression and Fast R-CNN train the identical training set and produce a detector in the experiment. In-order to obtain the assurance of the detected text, the extracted feature values of the relevant contour and target object contour is created first using the Deep Learning Network (DLN) with Fast R-CNN architecture. In-order to detect the same text and to get the associated feature point set, Gradient boost detector is deployed. The qualities of feature matching are assessed in accordance with the view of repeatability (repetition rate). The Eigenvalues which are extracted by the two algorithms are calculated. The contour region is determined by assigning the confidence level to 100% which matches the similar result. The Threshold value is fixed in the range 0 to 1. To identify the cyber-attack the matched pattern which are greater than the 0.5 threshold values are termed as “ATTACK” and less than or equal to 0.5 values are termed as “NORMAL”. The hybrid GBR model continues to get the analysis if the DLN suggestion is class 2 of anomaly. Based on the feature mappings already made and stored as.MAT file with the backend model, the classification of the abnormality of intrusions are categorized.

#### The RoI pooling layer

One must understand the Sub-Sampling Ratio before beginning the Region of Interest (RoI) Projection. It is the proportion of the feature map’s size to the image’s original size. The concept behind RoI projection is that we need to project the RoI proposal with regard to the subsampling ratio in order to obtain the bounding box coordinates from the RoI proposal and place them on the feature maps. We represent the co-ordinates of bounding box in 2 ways.

1. Co-ordinates for the box’s midpoint is (A,B) and width and height are represented as (X,Y)
2. Co-ordinates on the bounding box’s opposite side is represented as [A1,B1 & A2,B2].

In our proposed work we need 2D output since our model is a pre-trained network. It may not be possible to divide them equally, if the dimensions are odd numbered. So we are rounding the value to the nearest number. Now, the height and width are divided to form a fixed dimensional box as per the required data and rounding them to the nearest value. The output is calculated after max-pooling each block. We proposed [6x6] grid pooling in Fast R-CNN, because our proposed model utilizes “ALEXNET” architecture. Our Fast R-CNN model is 146 times faster than the existing one and it is very efficient in solving bounding box regressor’s L2 loss, SVM and Log loss.

#### Fine tuning for detection

To design our proposed NIDS model very efficiently, we need to be very careful in choosing the hyper-parameters. Our proposed model uses “Regression Learner app” for selecting the hyper-parameter values which are present in the hyper-parameter optimization blog. Using an optimization strategy that aims to reduce the model MSE, the app experiments with various combinations of hyper-parameter values and then returns a model with the optimized hyper-parameters.

First, we have to choose the best optimizer values. To search the corresponding data points in the dataset, iterations must be matched. Among the two types of “Grid Search” and “Random Search”, my proposed work uses the “Random Search” method with matching iterations of “1000 Epochs”.

We suggest a training strategy that is more effective and makes use of feature sharing. Stochastic gradient descent (SGD) mini-batches are hierarchically sampled in Fast R-CNN training. “N” data points are sampled first and the each data from R/N RoI’s are then sampled. “Memory” and “Computation” for the backward and forward passes are shared by RoIs from the same data. Mini-batch computation is decreased when “N” is made small. In our proposed work we use N = 2 and Mini Batch size as 128 which made my model 64 times faster. Even though the training time is high because of data correlation, we used SGD method which achieves better results than the existing methods. Instead of training a SVMs, regressors and Softmax classifier, in three different phases, they both are combined in a single stage of Fast R-CNN during the training process [38, 39].

#### Multitasking loss

A Fast R-CNN has two interconnected output layers. Layer 1 computes a discrete probability distribution from each RoI, That is k = (k0, k1,……..,kN), over N + 1 categories. The conventional method for calculating “k” is to use a Softmax over the N+1 outputs of a fully connected layer. The second sibling layer provides bounding-box regression offsets for each of the N object classes, indexed by r, as follows: t^r^ = (t^r^_x_, t^r^_y_, t^r^_w_, t^r^_h_). Here t^r^ specifies the log-space height/width shift and scale-invariant translation which are related to object proposals. Here “X” and “Y” are labelled as “ground-truth class” and “ground-truth bounding box regression target” for every one RoI. In-order to train the said label, we utilized a multitasking loss “M” on every labelled RoI.

$$M(k,v,t^{n},s)=M_{cls}(k,v)+\gamma [v\geq 1]M_{loc}(t^{n},s)$$ (17)

where, M_cls_(k,v) = -log k_v_ is true class log loss of “X”. M_loc_, the next loss is specified over a pair of real target class “X” for bounding-box regression. s = (s_x_, s_y_, s_w_, s_h_), and a predicted tuple t^n^ = (t^n^_x_, t^n^_y_, t^n^_w_, t^n^_h_) again for class “X”.[v ≥ 1] calculates to 1, or else 0. All background classes are labelled as X = 0. Since there is no concept of a ground-truth bounding box for background RoIs, L_loc_ is disregarded. Loss function used for bounding-box regression is,

$$M_{loc}(t^{x},v)=\sum_{i\in \{x,y,w,h\}}smooth\;L_{1}(t_{i}{}^{x}-V_{i})$$ (18)

in which

$$Smooth\;L1(S)=\{\begin{array}{l} 0.5S^{2}\;if\;|S|<1\\ |S|-0.5\;Otherwise \end{array}\}$$ (19)

Exploding gradients should be prevented when there are unbounding regression targets. We should be very careful when we tune the learning rates during L_2_ loss training. Losses between the two tasks are balanced by controlling the hyper-parameter “γ” in Eq 15.

To achieve “Mean = 0” and “Variance = 1” the ground-truth regression targets “Y” is normalized [38].

#### Batch size and epochs

Our proposed method uses 1000 training samples with batch size as 128. The proposed algorithm takes the 1^st^ 1000 samples (that is from 1 to 1000) from the training dataset to train the network. Again the process repeats for the 2^nd^ 1000 samples (that is from 1001 to 2000) to train the network. This process is repeated until all the samples are extracted from the dataset. The biggest advantage is it takes only less memory to train the model. Each time when we update the weights, the model is trained fast which will be an added advantage.

The one hyper-parameter which we should observe is nothing but an “Epochs”. Our proposed algorithm walks throughout the training dataset for the defined epochs. Our model is trained and tested with a minimum of “5” and a maximum of “1000” epochs. Our internal model parameters gets updated in the training dataset for every “1” epoch (that is for each sample) [40].

#### Stochastic Gradient Descent (SGD)

The most common optimizer algorithm which is proposed in our work is SGD. This algorithm optimizes the “learning rate” and “momentum”. The weights are controlled by the learning rate at the final stage of each batch, while the momentum updates the current weight which is influenced from the previous update. Our proposed model takes the “learning rate value as 0.0001” and “momentum value as 0.9” [39].

SGD can be calculated by using the below mentioned formula,

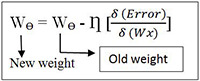

(20)

Here, “Ƞ” represents the → learning rate

“δ (Error)” represents → the derivative of error with respect to weight.

#### Softmax classifier

The features which are extracted finally from the hidden layer nodes are then given to “Softmax” classifier section which is placed at the end. Then they are categorized accordingly based on the pattern generated. This proposed classifier uses “One-Hot encoding to calculate the “Cross-Entropy Loss” and gain the maximum value. A true distribution “p” and an estimated distribution “q” have the following cross-entropy:

$$H(p,q)=-\sum_{x}p(x)\log q(x)$$ (21)

To minimize the cross-entropy between the true distribution and the predicted class, Softmax classifier seeks all the probability mass in the correct class. Then our proposed model should identify the correct object (cyber-attack) using the cross-entropy function. When the prediction is more accurate, the loss function is reduced.

#### Deep Inspection Packet (DPI)

A type of “Packet Filtering” method which is implemented in our proposed work to capture the anomaly packet. This method uses the technique called “Pattern Matching” as key for security in our proposed IDS model. This technique will decide the packets that must be allowed. One of the effective methods in identifying the “Anomaly packet” and in detecting the cyber-attacks. (Specially in DoS and U2R attacks).

#### Experimental setup

The performance of binary and multiclass classification are evaluated using the trained NIDS dataset, which is centered on our Fast R-CNN model, and is used to assess. According to the results of research, the majority of more than 99 percent of scenarios are true. Algorithm 1 describes our proposed model’s training process.

**Algorithm 1: Hybrid Deep Learning**

Procedure: Training

*Preprocessed NIDS train →Training Dataset*

*Preprocessed NIDS test → Testing Dataset*

*Feature extraction → PCA Values*

*Matrix decomposition → SVD Values*

*Compute the values for “X” → Gradient Boost Regression*

*i ← 0*

*Epoch ← 50*

*While i ≠ epoch* ***do***

*For each epoch repeat the training procedure*

*“Y” → Result is stored*

*Analyze the bias result and compare it to X*.

***if*** *testing accuracy is 0 (or) i = 0 then*

*Testing Accuracy → Best testing Accuracy*

***end if***

The suggested algorithms must be conducted in a stable environmental setting throughout the training and testing phases. The utilized software and hardware setups are displayed in the Table 3.

**Table 3:** Environmental setup.

**Operating System**	Microsoft Windows 11
**Tool Used**	MATLAB R2017b
**Processor**	Intel Core i7-8550
**Memory Speed**	1.6 GHz with Turbo Boost Upto 3.4 GHz
**RAM**	12GB
**GPU**	AMD Radeon 530 4 GB Graphic Card

In-order to prevent the data present in the computer system and IoT devices from external cyber threat, we need a best tool that protects the data efficiently. Many software tools are available for doing research work. Comparatively, “MATLAB” tool plays a vital role when it comes into the picture. Using MATLAB “Code Window” and “Simulink” we designed our proposed NIDS in an effective manner. Some of the key-points are shown below to develop our NIDS.

- Security → Design modelling and Code generation is secured.
- Analysis → Look for “Vulnerabilities” and “Compliance” in the code.
- Detection → “Cyber-Attack” detection at an early stage
- Performance → “Architecture” model can be checked.
- Design confidence increased when we use analytical methods.
- Reports → Address new risks after bug reports.
- Countermeasures → Defense mechanism can be added.
- Verifications → Automatic “Updates” and “Security checks” can be done.

During the testing phase, the Accuracy, Precision, Recall and F1-score evaluation measures are combined to create a performance grade for the deep learning model.

### Comparison scenario with proposed work of PCA SVD Fast R-CNN-GBR

#### Scenario– 1

Most of the existing IDS models fail in detecting cyber-attacks because of their high False Positive Rate (FPR) and high False Negative Rate (FNR). Hackers, virus and malwares become more risky for Computer networks nowadays. When developing an efficient IDS, Detection Rate (DR) should be considered as one of the main parameters. “Adaptive ability” should be considered as another parameter when developing an IDS. Poor lagging and failing are noticed in many existing models when a new intrusion behavior happens in the network.

Nowadays, huge amount of data are utilized for processing in various application fields, especially in the field of Image processing. As a result, “Big Data Analytics” came in light which can access large amount of data in a short span of time. Traditional IDS fails to identify any abnormal behavior at this place. This becomes a great challenge for an efficient IDS [41].

Our proposed model has high True Positive Rate (TPR) and less FPR. It also has the ability to withstand large dataset with high DR.

#### Scenario– 2

As data becomes more prevalent online, new options for attacks to target sensitive data have emerged, posing numerous security concerns. Among the 5 types of cyber-attacks, DDoS is considered as a major threat for computer network field. Most of the existing IDS are in-effective in handling high speed data. However traditional IDS performs well only with less amount of data with slow speed. Moreover time consumption is too high in detecting the DDoS attack. So when developing an efficient IDS, the model’s speed and time consumption should also be considered as main parameters [42].

Our proposed work consumes only 9.19 seconds in detecting the DDoS attack. Hence our proposed model is efficient and it outperforms in the above said issue.

#### Scenario– 3

To build a complete anomaly-based Network Intrusion Detection System (A-NIDS), hybrid method plays a crucial role. Here, Glowworm Swarm Optimization-Principal Component Analysis (GSO-PCA) is hybridized to enhance the performance of the IoT. So detecting the anomaly pattern efficiently will increase the performance of the model [43].

Our proposed model is developed by hybridizing Fast R-CNN with GBR to increase the performance of the model. We also proposed PCA technique to extract the features from the dataset. Our proposed method consumes less time in detecting the “Anomaly attack” with an overall performance of above 99% which outperforms the existing methods.

### Performance evaluation

Our proposed model detects the cyber-attacks as “Anomaly” (or) “Normal” which is trained and tested using NIDS dataset V.10 2017. The performance of the proposed system was evaluated using Mean Square Error (MSE). Lesser the value, higher the performance. Precision and Recall calculates the success of the prediction attack, while F-measure calculates the harmonic mean of Precision and Recall. A good IDS can help to achieve high DR with low FAR by identifying and classifying anomalous instances correctly. The model’s performance shows the best results when it uses deep learning technique which outperforms other existing methods.

#### Accuracy

The accuracy ratio is calculated by taking into account the number of attacks that have been correctly classified against the number of attacks that have been counted incorrectly.

$$Accuracy(A)=\frac{TN+TP}{TN+TP+FN+FP}$$ (22)

Where “True Negative” *(TN)* represents a situation in which there has been no attack and there has been no detection. While “False Negative” *(FN)* represents when an attack occurs but no alarm is sounded.

#### Precision

The precision measure is used to analyze the fraction of test data that is considered an attack.

$$\mathrm{Precision}=\frac{TP}{TP+FP}$$ (23)

Where “True Positive” *(TP)* is a real attack that sets off an alert. While “False Positive” *(FP)* represents a signal that sets off an alert even though there hasn’t been an attack.

#### Recall

The term recall is used to describe a sensitivity or true positivity. The terms recall and sensitivity are often interchangeably used to describe true positivity or sensitivity.


$$\mathrm{Recall}\;(r)=\frac{\mathrm{Sum}\;\mathrm{of}\;\mathrm{relevant}\;\mathrm{attack}\;\mathrm{detected}}{\mathrm{Total}\;\mathrm{sum}\;\mathrm{of}\;\mathrm{relevant}\;\mathrm{attack}\;\mathrm{in}\;\mathrm{the}\;\mathrm{database}}$$ (24)

#### F-Measure

The F-measure is a type of harmonic mean that is composed of the combination of recall and precision,

$$F‐\mathrm{measure}=\frac{2pr}{p+r}$$ (25)

#### Mean square error

The square of the difference between actual and predicted values is referred to as the mean or average.

$$MSE=\frac{1}{n}=\times \Sigma \;{\left(\mathrm{actual}-\mathrm{predicted}\right)}^{2}$$ (26)

Where “n” is the sample size.

## Experimental results

### PCA and SVD results

**Fig 6:**
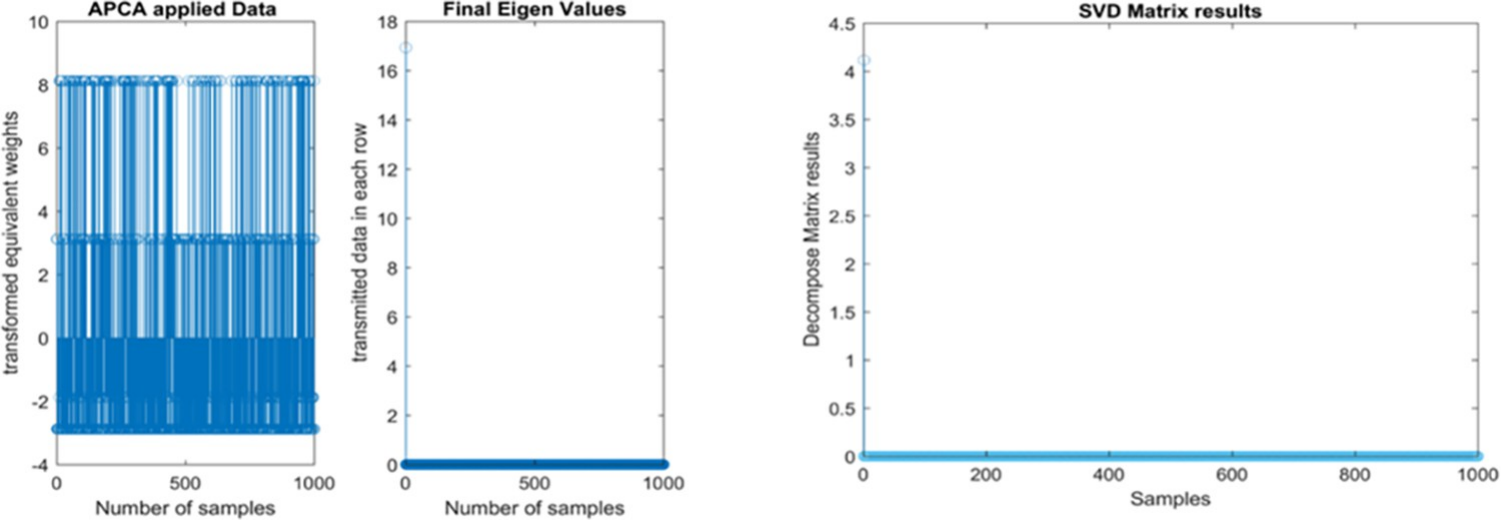
**(a).** PCA and eigen values of proposed model. **(b).** SVD matrix results of proposed model.

From Fig 6(A), it can be clearly seen that a new dataset defines the new variables, and finding such new variables, the principal components, is reduced by solving an eigenvalue / eigenvector problem. The matrix dimension is reduced by setting up the singular values that approach “0” which are smaller vales are shown in Fig 6(B). The final values are set to “0’s” by removing the unwanted columns.

#### Gradient Boost Regression results

**Fig 7:**
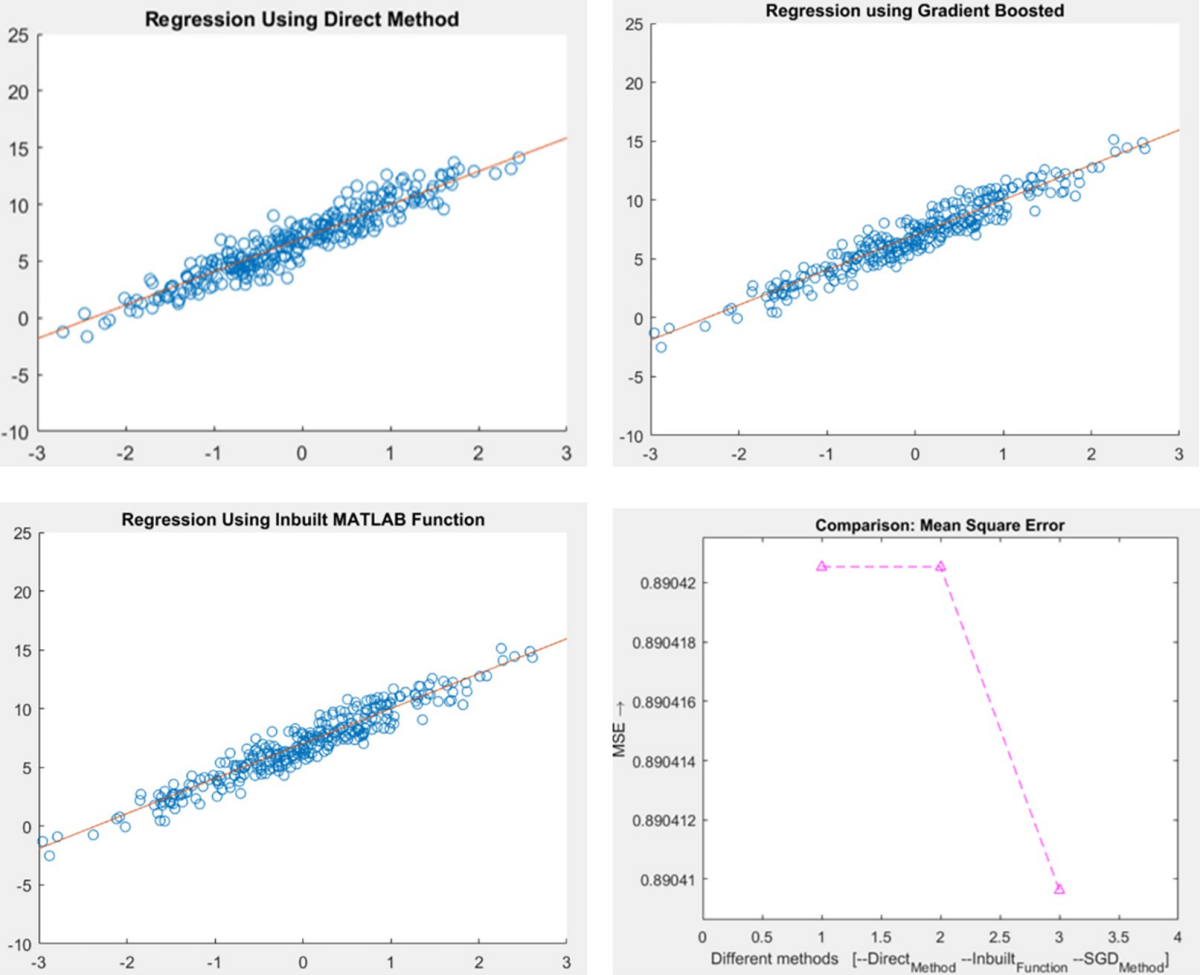
**(a).** Proposed result using direct method. **(b).** Proposed result using gradient boost regression. **(c).** Proposed result of hybrid method using In–Built MATLAB. **(d).** Proposed result of mean square error (MSE).

Fig 7(A)–7(C) show the results of “Direct method (Fast R-CNN)”, “Gradient Boost Regression” and “Hybrid Model” respectively of Buffer Overflow attack. The intrusions which are fetched from the proposed Fast R-CNN model (i.e., after PCA and SVD) and the feedback considered from the test input are given into the Gradient Boost Regression (GBR) model to analyze the relative correlation. Higher the correlation with the training anomaly considering Smurf, Neptune, Guess Password, IP Sweep, Buffer Overflow and Normal higher the formation of class.

Fig 7(D) shows the “Mean Square Error (MSE)” of the proposed model. It is computed by finding the “Mean” or the “Average” of the squared errors from data related to the function. Our model shows that the “Regression Line” is very close to the data set. By reducing the “Mean Square Error” values, the performance of the model increased [44].

**Fig 8:**
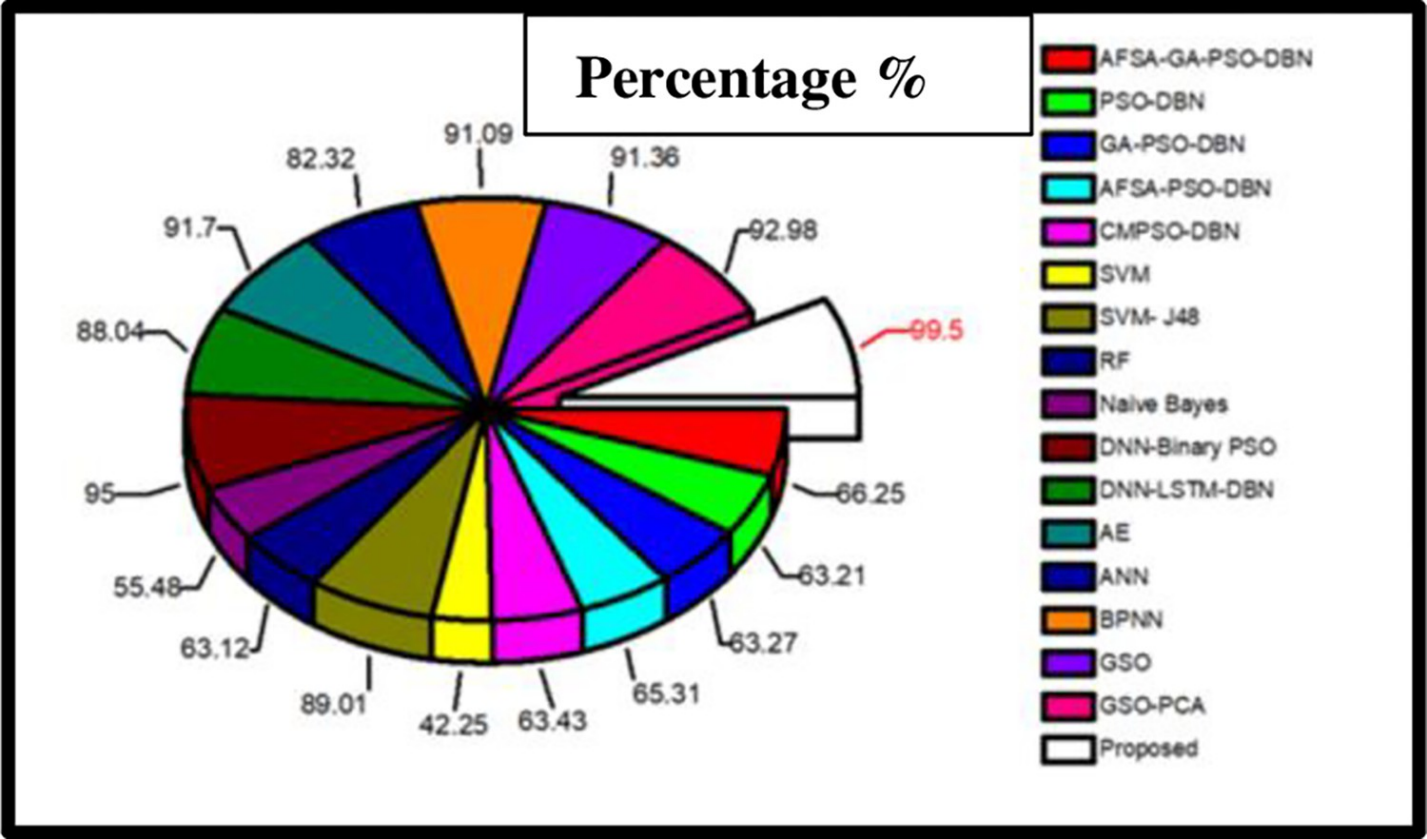
Overall accuracy comparison for different algorithms.

Fig 8 shows the overall “Accuracy” comparison result with various algorithms for our proposed model. “Artificial Fish Swarm Algorithm-Genetic Algorithm-Particle Swarm Optimization-Deep Belief Network (AFSA-GA-PSO-DBN), PSO-DBN, GA-PSO-DBN, AFSA-PSO-DBN, Crossover Mutation Particle Swarm Optimization (CMPSO-DBN), SVM, Random Forest (RF) AND Naive Bayes algorithms has very less percentage of accuracy in detecting the cyber-attacks. Algorithms like SVM-J48, DNN-LSTM-DBN, and Artificial Neural Network (ANN) has accuracy percentage of 89.01%, 88.04% and 82.32% respectively, which are slightly better than above said algorithms. DNN-Binary-PSO, AE, Back Propagation Neural Network (BPNN), GSO, Glowworm Swarm Optimization-Principal Component Analysis (GSO-PCA) have accuracy percentage of 95.00%, 91.7%, 91.09%, 91.36%, and 92.98% respectively, which are even better than the other existing algorithms [45, 46].

Finally, our proposed hybrid model has the highest accuracy percentage of 99.5% which outperforms all other algorithms. Hence the above graph clearly proves that our model is best in detecting the cyber-attacks efficiently.

**Fig 9:**
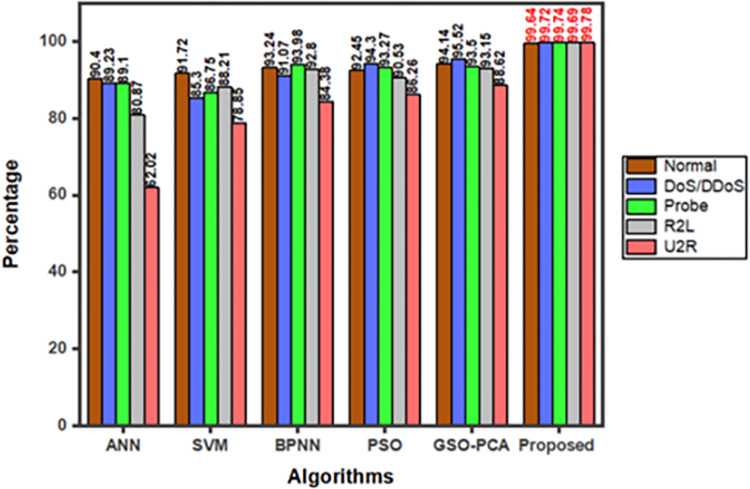
Accuracy comparison for individual cyber attack with various algorithms.

Fig 9 compares the individual cyber-attacks for accuracy results with other methods such as ANN, SVM, BPNN, PSO and GSO-PCA against four types of attacks namely DoS/DDoS, Probe, R2L and U2R attacks [42].

In all cases, proposed hybrid Fast R-CNN-GBR framework attains higher accuracy of more than 99.64%, 99.72%, 99.74%, 99.69% and 99.78% for the above-mentioned cases which are comparatively higher than the existing algorithms.

**Fig 10:**
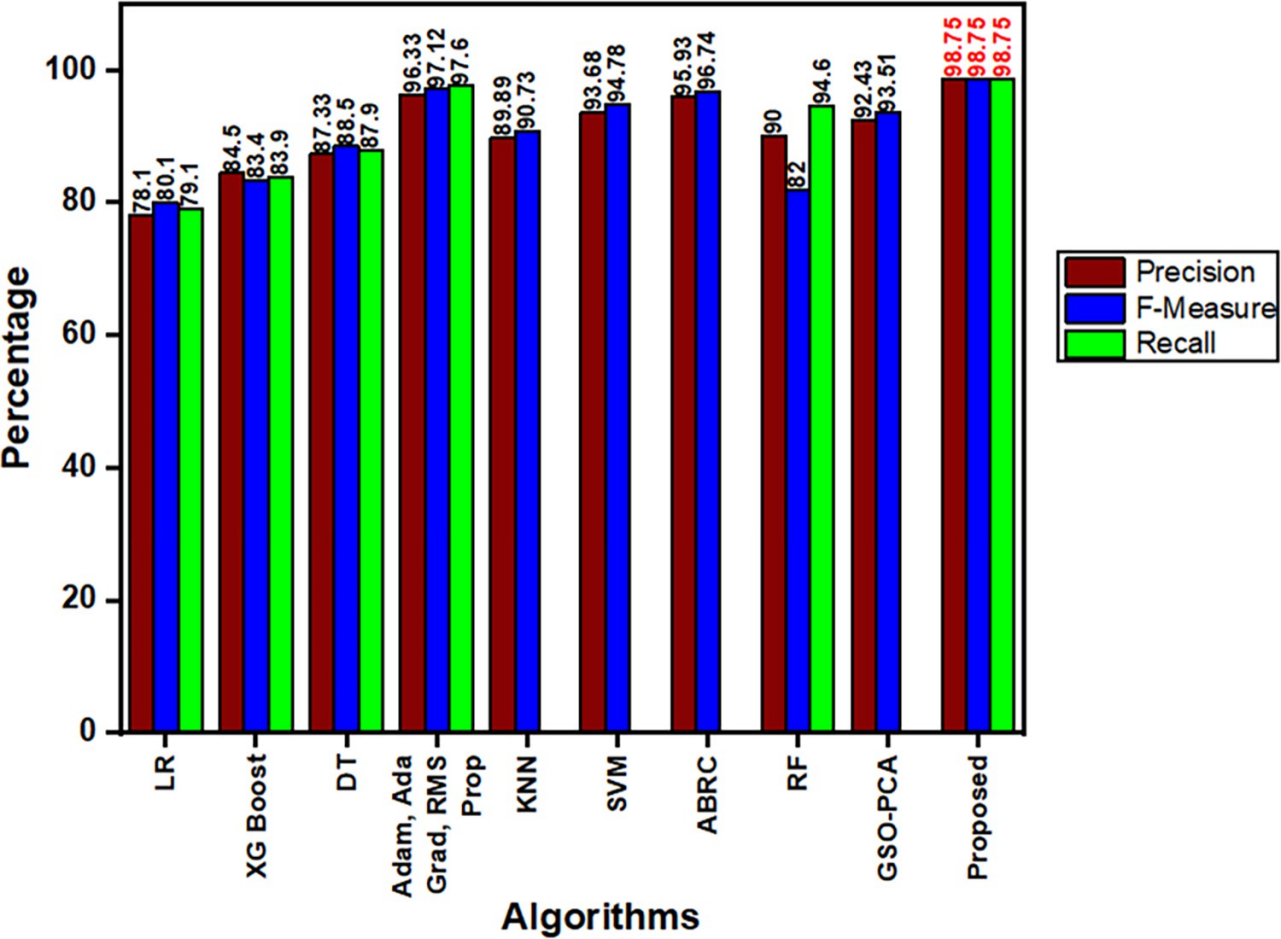
Performance metrics comparison of various algorithms.

Fig 10 shows the performance metrics comparison of Precision, Recall and F-Measure for various algorithms which are compared with our proposed work. From the figure, Logistic Regression (LR) shows very less performance when compared to other algorithms. Extreme Gradient Boosting (XG Boost), Decision Tree (DT) and K-Nearest Neighbor (KNN) performs slightly better than LR and Random Forest (RF’s) Recall value shows only 82% which is less.

Hybrid methods like Adam, Adaptive Gradient (Ada Grad) and Root Mean Squared Propagation (RMS Prop), SVM, Ada Boost Regression Classifier (ABRC) and GSO-PCA shows high performance in all the three parameters which overcomes other methods [42, 47–49].

Finally our proposed hybrid model shows the best overall performance in all the three metrics (Precision, Recall and F-Score) of about 98.75% which outperforms all other existing algorithms.

**Fig 11:**
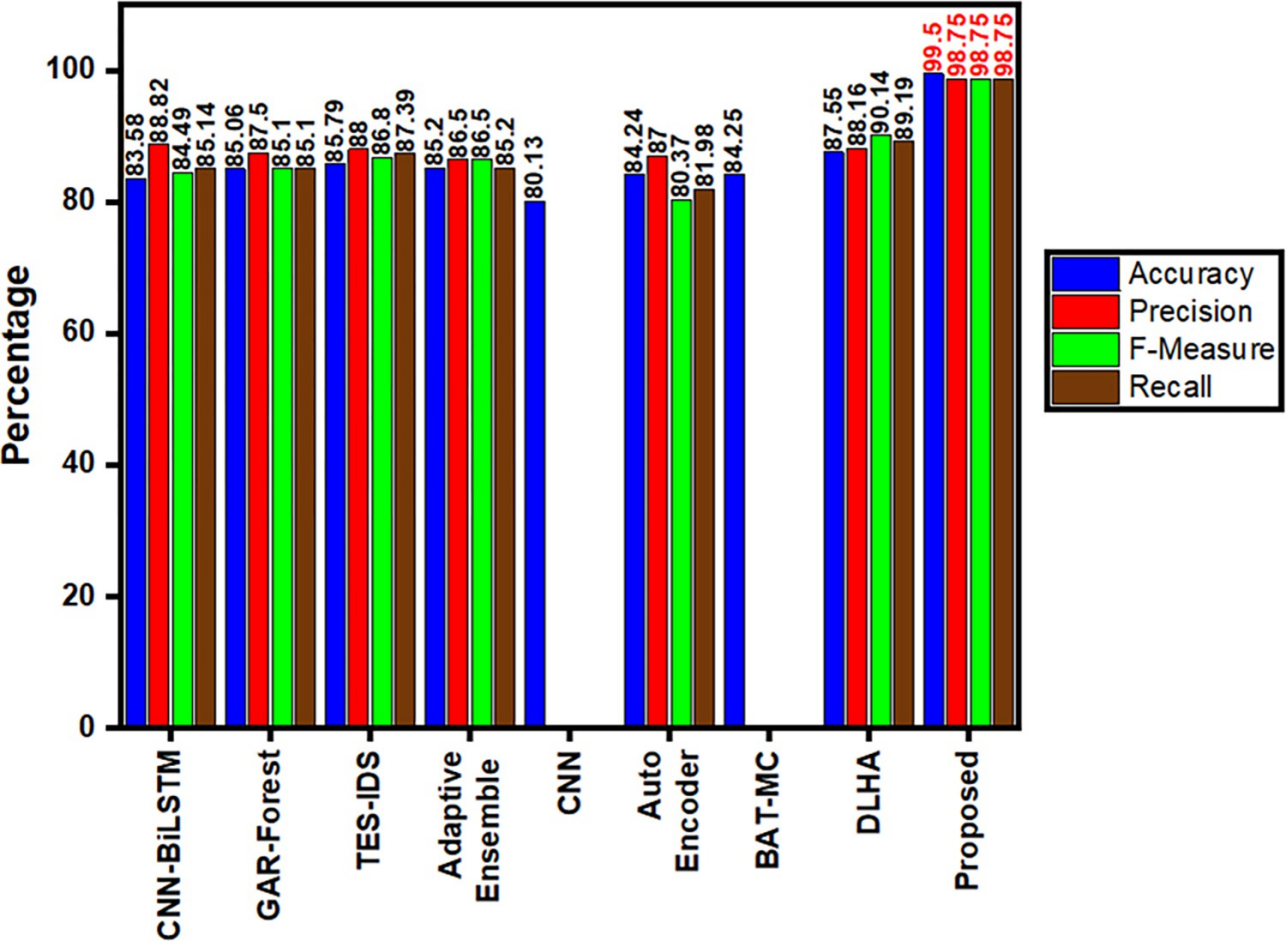
Performance comparison analysis.

In Fig 11, the proposed model shows that it outperforms other network models in terms of its performance. It also performed well in terms of its F1 score and accuracy. The proposed model does not require the use of manual feature extraction, which is typically required for traditional methods such as Greedy randomized Adaptive search procedure with Annealed Randomness (GAR-Forest) or Naïve Bayes (NB) Tree. It can improve the accuracy and reduce the need for manual intervention. The proposed model was able to achieve better classification results than the AE when it was compared with CNN.

It was first able to extract the data from the various features of the network traffic. The proposed model takes into account the various features of the network and re-assigns the channel weights according to their relationship with the other features. The proposed PCA SVD Fast R-CNN-GBR model achieves an accuracy of 99.5%, and Precision, Recall and F-Measure values as 98.75%. It is widely used for developing network security systems and it outperforms other reference models [50–57].

**Fig 12:**
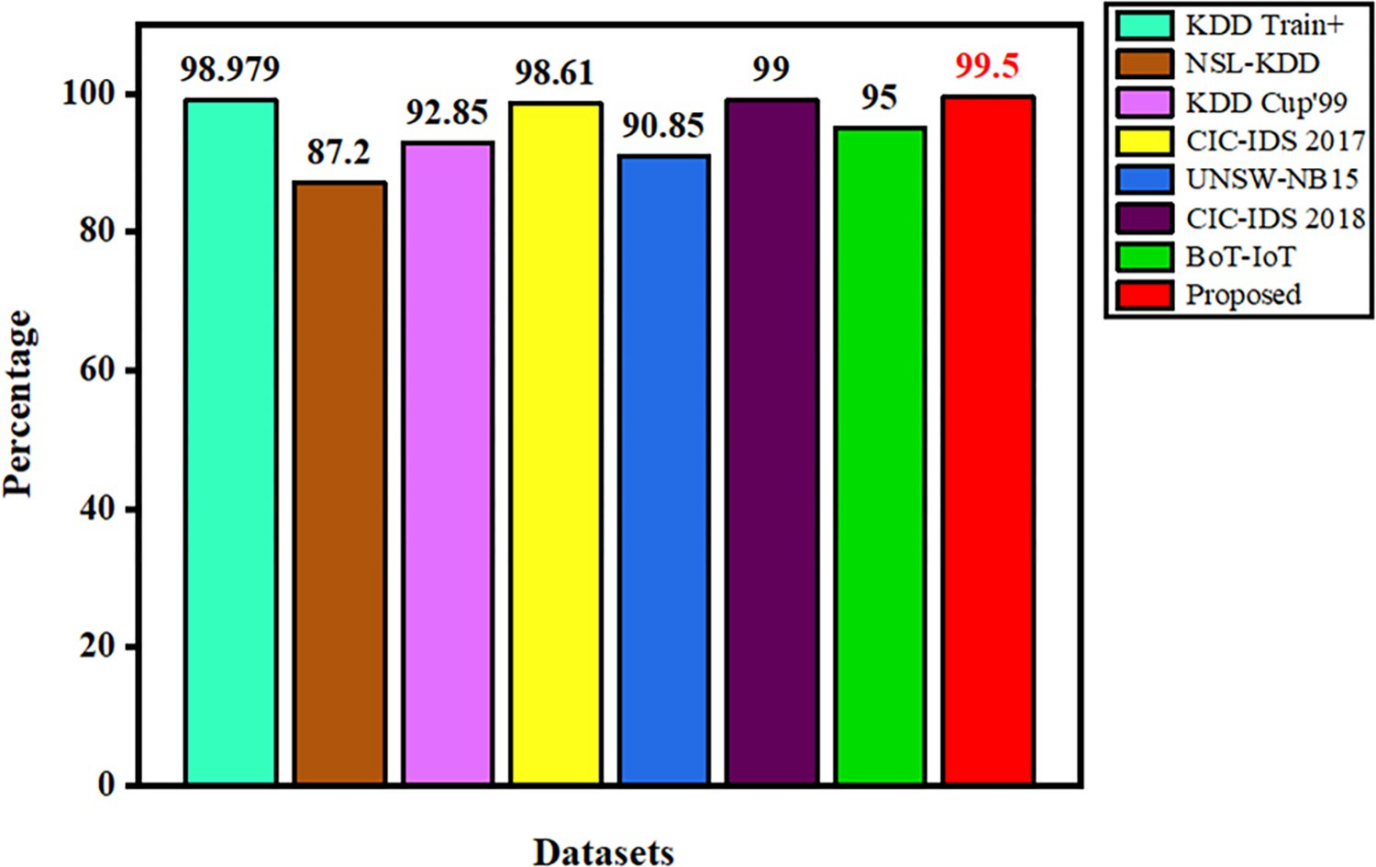
Accuracy comparison with various datasets.

The accuracy comparison for various datasets are compared with our proposed NIDS dataset V.10 2017 as shown in Fig 12. From the figure the NSL-KDD dataset shows less detection accuracy of only 87.2% when compared to other datasets. UNSW-NB15, KDD Cup’99 and BoT-IoT datasets overcome the previous mentioned datasets in detecting the cyber-attacks ranging from 90% to 95% respectively. The CIC-IDS 2017 and KDD Train+ datasets surpasses the other datasets in detecting the cyber-attacks with 98% accuracy level. After choosing hyper-parameters carefully and fine tuning the model, our proposed method outperforms other all datasets in detecting the cyber-attacks, and hence our proposed model is efficient when compared to other existing methods [25, 58–61]

## Discussion

Fast R-CNN a Deep Neural Network which has been discussed is said to be very trustworthy and quickly detects and categorizes the cyber-attacks. On successfully combining Gradient Boost Regression with Fast R-CNN the proposed model minimizes False Positives, increases maximum accuracy, and simplifies computation complexity. The correctly detected cyber-attack output by Gradient Boost Regression is shown in Fig 7(A)–7(C). The results once again proved that the proposed model is very efficient in detecting the cyber-attacks. “Accuracy” is one of the most important parameters to be discussed. Since the proposed model uses “Regression” tactics, the models accuracy is based on MSE. Fig 7(D) shows the MSE output, from which it is known that when MSE decreases the model’s accuracy increases. The obtained accuracy is shown in Fig 8. With various hyper-parameter tunings and settings, the proposed model is tested for various epochs, in-order to increase the performance. The multiclass classification result which is shown in Fig 9 are compared with various algorithms. It is also necessary to compare the performance metrics with other existing algorithms. Figs 10 and 11 show that the proposed hybrid Fast R-CNN with Gradient Boost Regression outperforms other algorithms in-terms of performance in a very short amount of time. It is also important to show that our proposed dataset which is used in our proposed model shows best detection accuracy when compared to other datasets. So once again we claim that our proposed model is best in all aspects and the corresponding figures prove that.

## Conclusion

This work has extensively summarized the applicability of Fast R-CNN in NIDS. Our proposed method results are compared with various other algorithms. NIDS V.10 2017 dataset is openly available in KAGGLE website which is used as a prominent tool for the research work. The collected dataset is split into training and testing. In order to increase the effectiveness of attack detection, the data are trained using various layers. From the results we clearly establish that, compared to other algorithms the proposed method proves best in terms of “Accuracy”, “Precision”, F-Measure” and “Recall”. In order to further improve the technique, this work considers hybridizing Fast R-CNN and Gradient Boost Regression with various numbers of hidden layers, and it was shown that Deep Neural Network (DNN) with two layers is the most efficient and accurate of them. We can infer from the empirical findings of this research that deep learning techniques is a viable route for cyber security.

## Challenges, limitations and future scope

The major challenges behind the cyber-attacks depend on various activities of the network channel within the given time. The key factors like complexity, database update, stability and limberness are still great challenges for IDS. The generation of false alarm that matches with normal activity of the system is one of the challenges in prediction. Although the performance on an imbalanced dataset is excellent, it is still important to apply the same techniques to real-time network traffic that incorporates more advanced and modern attack types. Further research is needed to determine how adaptable these DNNs are in real-time situation. An efficient IDS should be evaluated rigorously, since the “Deep Learning” technology which utilizes diverse algorithms are escalating very fast. Reducing the “Propagation Delay” is also one of the important tasks because of the data collected by the IoT. This will be one of the possible directions for IDS research and will therefore continue to be a work in progress.
